# Thyroid Cancer in Kazakhstan: Component Analysis of Incidence Dynamics

**DOI:** 10.31557/APJCP.2019.20.9.2875

**Published:** 2019

**Authors:** Nurbek Igissinov, Saken Kozhakhmetov, Marzhan Zhantubetova, Gulnur Igissinova, Zarina Bilyalova, Gulnur Akpolatova, Vladimir Lust, Sarsenbi Koblandin, Dulat Turebayev, Kairat Adaibayev, Ardak Omarbekov, Dinara Tarzhanova, Akmaral Zhantureyeva, Askar Esseyev

**Affiliations:** 1 *Astana Medical University,*; 3 *Central Asian Cancer Institute, Nur-Sultan,*; 5 *National Medical University, Almaty, Kazakhstan,*; 2 *International High School of Medicine,*; 4 *Eurasian Institute for Cancer Research, Bishkek, Kyrgyzstan. *

**Keywords:** Thyroid Cancer (TC), incidence, component analysis Kazakhstan

## Abstract

**Background:**

The International Agency for Research on Cancer (IARC) reports that 567,000 new cases of thyroid cancer (TC) were registered in the world in 2018, and the age-standardized incidence rate was 6.7 per 100,000. The Global Cancer Observation forecasts a 35% growth in the number of new cases worldwide by 2040. The number of patients with TC in Kazakhstan is also increasing steadily. This investigation was the first epidemiological study of TC trends by component analysis among the population of Kazakhstan. This paper presents the results of the component analysis of TC incidence trends in Kazakhstan.

**Methods:**

The study covers primary data of TC cases (ICD 10 – C73) registered throughout Kazakhstan from 2009 to 2018. TC incidence trends were evaluated using component analysis according to the methodological recommendations.

**Results:**

5,559 new TC cases were registered during the 10-year study period. The average age of patients was 52.0**±**0.2 years, the average annual age-standardized rate in 2009-2018 was 3.3**±**0.2^0^/_0000_, with a constant upward trend (Т=+6.6%). According to the component analysis results, the increase in incidence was mainly due to the combined effect of the two factors: the increased disease risk (Δ_R_=+61.7%), and the population growth (Δ_P_=+15.4%).

**Conclusion:**

The noted increase in incidence was mainly caused by the changes in risk factors, such as the worsening environmental aspects and the increase in detection of clinically non-manifesting cases. The results of the study shall be taken into account when planning anticancer activities for TC.

## Introduction

Epidemiological studies of thyroid cancer (TC) indicate significant changes in its incidence rates over the past three decades. TC ranks 9^th^ among all malignant neoplasms, which is 3% of all cancer cases. According to the Global Cancer Observation, by 2040 the annual number of new TC cases will increase by 35%, from 567,233 to 764,573 worldwide (Ferlay et al., 2018A; Bray et al., 2006; Ferlay et al., 2019). Geographic variability and heterogeneity of TC incidence are linked to various exogenous and endogenous factors, including the epidemiological situation in the region (Muhammad Aleem Khan et al., 2016). Radiation epidemiology data based on the long-term study of radiation impact on the survivors of the atomic attack in Hiroshima and Nagasaki clearly demonstrates that the increase in the risk of TC as a result of external exposure is dose- and age-dependent (Yamashita et al., 2018). A sharp increase in TC incidence in children was reported in Belarus four-five years after the Chernobyl accident (Kazakov et al., 1992; Zablotska et al., 2015; Cahoon et al., 2017). The dose-dependent risk of TC and the results of in-vitro irradiation experiments show that the exposure to high doses causes genetic changes, such as gene rearrangements observed in TC tissues. So, irradiation is a clear risk factor for TC (Furukawa et al., 2013; Veiga et al., 2016). In addition, the environmental situation in the region also affects the TC incidence. The regions with a high TC incidence include such American and European countries as Canada (19.5^0^/_0000_), USA (14.5^0^/_0000_), France (14.2^0^/_0000_), Croatia (12.1^0^/_0000_), Portugal (11.7^0^/_0000_), Greece (10.3^0^/_0000_), and others. The highest TC incidence in the world is observed in Korea – 60.7^0^/_0000 _(La Vecchia et al., 2015). In other countries, the TC incidence varies widely (Ferlay et al., 2018B).

Purpose of this article is to characterize the sources of cancer patients’ data and the factors that determine the completeness and accuracy of data, as well as to present the technique of statistical evaluation of TC incidence trends among Kazakhstani population by methods of component analysis. Such analysis for TC has never been done in Kazakhstan before. Such analysis of changes of certain factors affecting the TC incidence will help the cancer care providers to identify the causative factors and make cancer control in the studied areas of Kazakhstan more targeted. The analysis also allows the regional oncology managers to properly account for the TC incidence trends as an important indicator of public cancer care , as well as to assess the status of detection and the quality of diagnostics (Igissinov et al., 2012; Igissinov et al., 2013; Igissinov et al., 2015; Kuanyshkalieva et al., 2016).

## Materials and Methods

The study included a retrospective analysis of primary data of patients registered with malignancies all over the country. Average sex- and age-related figures for the studied years were taken from the official website of the Committee for Statistics of the Ministry of National Economy of the Republic of Kazakhstan (www.stat.gov.kz).

The study was approved by the local ethical committee. The information may be shared for research purposes only if a requesting organization ensures the data security and takes all the necessary actions to unable the identity of the respondents in concordance with the Declaration of Helsinki − Ethical Principles for Medical Research Involving Human Subjects, adopted by the 18^th^ WMA General Assembly in Helsinki, Finland, in June 1964, and amended by the 64^th^ WMA General Assembly, Fortaleza, Brazil, October 2013 (Declaration of Helsinki, 2013).

Descriptive and analytical methods of epidemiology were used in studying the TC incidence (dos Santos Silva, 1999). Extensive, age-specific, crude and age-standardized incidence rates (ASR) were calculated using the generally accepted methodology of biomedical statistics (Tango, 2010; Chissov et al., 2007). ASR values were calculated directly, using the world population standard (Omar et al., 2001) adjusted according to NCI SEER recommendations (NCI SEER recommendations, 2013). The incidence rates were calculated per 100,000 of the relevant population. The incidence trends were determined for 10-years periodby least squares method. The average annual growth/decrease of the dynamic range was determined by the geometric mean. The average age of patients, average values (M, P), average error (m), 95% confidence intervals (95% CI), and average annual growth /decrease of equalized indicators (T, %) were calculated.

The TC dynamics among the population of Kazakhstan was studied following the Dvoyrin and Aksel guidelines (Dvoyrin et al., 1987). This method of analysis allows segmenting the number of patients within the same population in different time periods. There are seven different components of growth of the number of cases: first three are related to the changes in population number, age structure of population (ASP), and the combined influence of these factors; the fourth is the change of risk index of the malignant thyroid tumor; the remaining three are related to the population growth risk, changes in ASP and the resulting effect of all three factors.

Many researchers consider the disease risk as a whole range of factors that can increase, reduce or stabilize the morbidity indexes. Therefore, the increase in disease risk corresponds to the latter four components.

The seven components are grouped into 3 classes. Class 1 represents different kinds of population changes (Δ_P_+Δ_A_+Δ_PA_); Class 2 shows the growth of the disease risk (Δ_R_); Class 3 represents the correlation between all factors (Δ_PR_+Δ_AR_+Δ_PAR_). Thus, factors from all the three classes shall be summed up to characterize the cumulative effect of population changes and the disease risk: 

1. (Δ_P_+Δ_A_+Δ_PA_)+(Δ_PR_+Δ_AR_+Δ_PAR_) 

2. Δ_R_+(Δ_PR_+Δ_AR_+Δ_PAR_) 

The component method was used to analyze the dynamics of TC incidence based on the number of cases that occurred from 2009 to 2018 throughout Kazakhstan. Mathematical calculations for the component analysis of TC incidence trends among the population of Kazakhstan were made using Microsoft Excel program. The results are presented in the attached tables. 

Symbols and abbreviations used in the article: ASR, age-standardized rate; Р, the thyroid cancer incidence rate; ASP, the age structure of the population; SI, structural indexes; RP, risk of progressing; NTC, the number of TC cases; PN, population number; ENC, the expected number of cases.

## Results

5,559 new TC cases were registered during the 10-year study period (2009-2018), of them, 786 cases (14.1%) in men and 4,773 cases (85.9%) in women. In both sexes, the incidence was growing unimodally, with a peak incidence at the age of 50-59 years (28.0% in men and 26.2% in women, 26.5% of all new cases). Age-related morbidity was growing in almost all age groups in the studied population, except for men of 60-69 years, where the trend was downward (T=−0.7%) (Table 1).

**Table 1:** The Average Annual Thyroid Cancer Incidence in Kazakhstan, by Age and Sex, 2009-2018

Age	All			Men			Women		
	Number (%)	Incidence		Number (%)	Incidence		Number (%)	Incidence	
		^0^/_0000_	Т, %		^0^/_0000_	Т, %		^0^/_0000_	Т, %
<30	487 (8.8)	0.6±0.1	6	69 (8.8)	0.2±0.02	5.8	418 (8.8)	1.0±0.1	6.1
30-39	782 (14.1)	3.1±0.3	9.4	86 (10.9)	0.7±0.1	3.8	696 (14.6)	5.3±0.5	10.2
40-49	1,084 (19.5)	5.0±0.4	8.9	142 (18.1)	1.4±0.1	8.9	942 (19.7)	8.3±0.8	8.9
50-59	1,472 (26.5)	8.0±0.5	4.2	220 (28.0)	2.6±0.3	5.5	1,252 (26.2)	12.5±0.7	4.2
60-69	1,096 (19.7)	10.8±0.7	6.2	172(21.9)	4.2±0.2	−0.7	924 (19.4)	15.4±1.2	7.7
70+	638 (11.5)	8.2±0.6	5.2	97 (12.3)	3.7±0.4	2.3	541 (11.3)	10.4±0.8	5.6
Total	5,559 (100.0)	3.2±0.2	7.3	786 (100.0)	0.9±0.1	5	4,773 (100.0)	5.4±0.4	7.8
ASR	−	3.3±0.2	6.6	−	1.1±0.1	3.8	−	5.1±0.4	7.2

**Table 2:** Component Analysis of Thyroid Cancer Incidence in Dynamics in Kazakhstan, 2009-2018

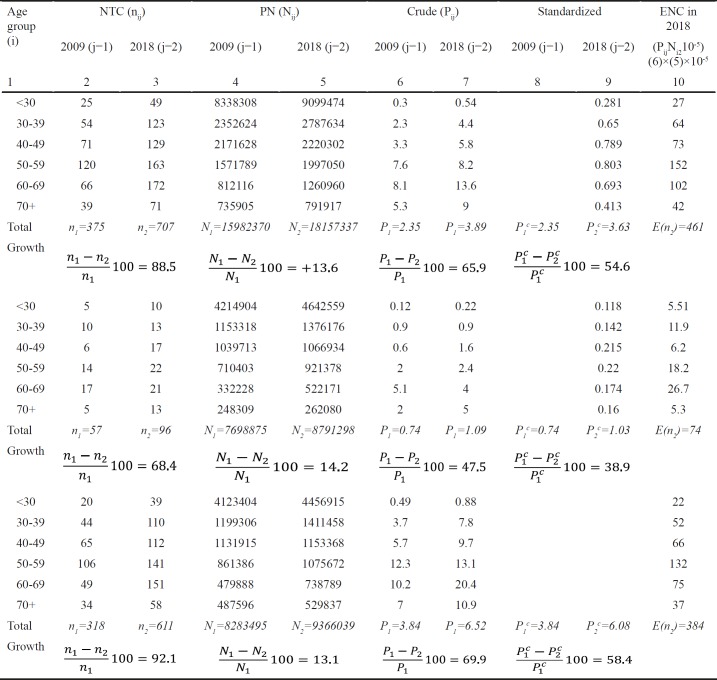

**Table 3:** Components, Influencing the Number of Thyroid Cancer Cases in Kazakhstan, by Sex

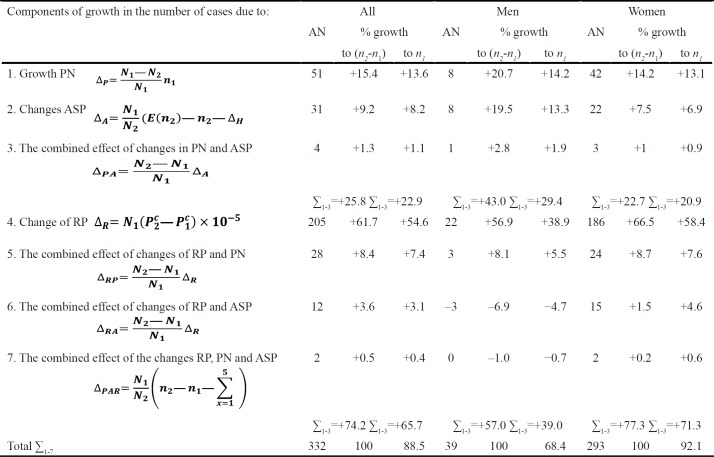

**Figure 1:**
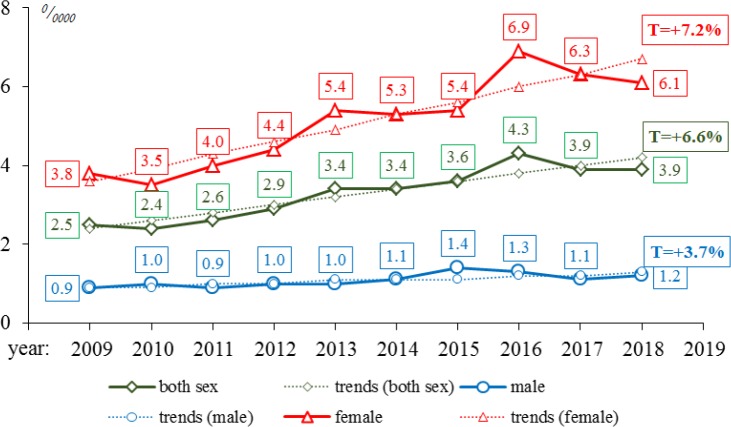
Dynamics of Age-Standardized Thyroid Cancer Incidence Rates (ASR), 2009-2018

The average age of patients was 52.0**±**0.2 years (95%CI=51.7-52.4) during the whole study period (T=−0.1%). At that, the average age of male patients was equal to 53.2**±**0.5 years (95%CI=52.3-54.1) and decreasing during the study period. The average age of female patients was a bit lower (51.8**±**0.2 years, 95% CI=51.5-52.2), and remained stable over time (T=−0.1%).

ASR during the study period has amounted to 3.3**±**0.2^0^/_0000_ (95% CI=2.9-3.7) with an upward trend in both sexes (T=+3.7% in men, T=+7.2% in women, T=+6.6% in both sexes). At the same time, ASR in men (1.1**±**0.05^0^/_0000_) was almost five times less than in women (5.1**±**0.4^0^/_0000_); the difference was statistically significant (t=10.44, p=0.00) (Figure 1).

Crude TC incidence rates for the total population has increased from 2.35^0^/_0000_ in 2009 to 3.89^0^/_0000 _in 2018. At that, the average increase has amounted to 1.550/0000 and was dependent on the changes in ASP (∑=Δ_A_=+0.19^0^/_0000_), the disease risk (∑=Δ_R_=+1.28^0^/_0000_) and the combined effect of changes in those parameters (∑=Δ_AR_=+0.07^0^/_0000_).

In men, crude TC incidence has also increased from 0.74^0^/_0000_ in 2009to 1.09^0^/_0000_ in 2018. The total increase (+0.35^0^/_0000_) was associated with the changes in ASP (∑=Δ_A_=+0.10^0^/_0000_) and the disease risk (∑=Δ_R_=+0.29^0^/_0000_), while the combined effect of changes in those parameters has led to a decrease in crude incidence rates (∑=Δ_AR_=−0.04^0^/_0000_).

In women, the total increase (+2.68^0^/_0000_) in crude incidence rates from 3.84^0^/_0000_ in 2009 to 6.52^0^/_0000_ in 2018 was dependent on the changes in ASP (∑=Δ_A_=+0.27^0^/_0000_), the disease risk (∑=Δ_R_=+2.24^0^/_0000_), and the combined effect of changes in those parameters (∑=Δ_AR_=+0.18^0^/_0000_)

Tables 2 and 3 present the component analysis of NTC dynamics in Kazakhstan from 2009 to 2018 in men, women and both sexes.

In general, the changes in the number of TC cases in Kazakhstan could be caused by the following factors (Table 3):

1. Growth in PN Δ_P_=+15.4% (Male - Δ_P_=+20.7%. Female - Δ_P_=+14.2%).

2. Changes in ASP Δ_A_=+9.2% (Male - Δ_A_=+19.5%. Female - Δ_A_=+7.5%).

3. The combined effect of changes in PN and ASP Δ_PA_=+1.3% (Male - Δ_PA_=+2.8%. Female - Δ_PA_=+1.0%).

4. Change in disease risk Δ_R_=+61.7% (Male - Δ_R_=+56.9%. Female - Δ_R_=+66.5%).

5. The combined effect of changes in disease risk and PN Δ_PR_- +8.4% (Male - Δ_PR_=+8.1%. Female - Δ_PR_=+8.7%). 

6. The combined effect of changes in disease risk and ASP Δ_AR_=+3.6% (Male – Δ_AR_= -6.9%. Female – Δ_AR_=+1.5%).

7. The combined effect of changes in disease risk, PN and ASP Δ_PAR_=+0.5% (Male – Δ_PAR_= -1.0%. Female – Δ_PAR_=+0.2%).

The total increase in the absolute number of TC cases in both sexes is a sum of all causative components:

n_2_−n_1_=51+31+4+205+28+12+2=332, or +88.5% to the baseline number of cases (332/375×100=88.5%).

At that, the components affecting the percentage of increase vs. the baseline level were similar for the whole population:

**Figure:**
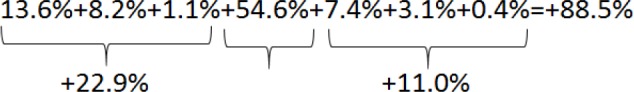


For the total population, the real increase in the number of cases was high (54.6%). However, only 22.9% out of 88.5% of the total increase was caused by the changes in PN.

The total increase in men has equaled to: *n*_2_*-n*_1_=8+8+1+22+3−3−0=39 or +68.4% compared to 2009 (39/57×100=68.4%). The components included:

**Figure:**
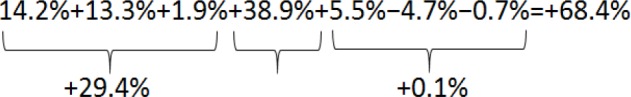


In men, the real increase in the number of TC cases was quite high (38.9%). However, only 29.4% out of 68.4% of the total increase in incidence in men was caused by the changes in PN.

The total increase in women has equaled to: *n*_2_*-n*_1_=42+22+3+186+24+15+2=293, or 92.1% compared to 2009 (293/318×100=92.1%).

The components included:

**Figure:**
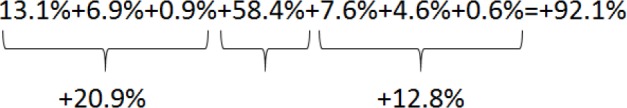


Thus, in women, the real increase in the number of TC cases was high (58.4%). However, only 20.9% out of 92.1% of the total increase in incidence in women was caused by the changes in PN.

## Discussion

The analysis of average ASR places Kazakhstan among the regions with medium incidence rates (ASR 3.3**±**0.2^0^/_0000_). Similar rates are observed in such countries of South-Western Europe as Serbia (3.8^0^/_0000_), Armenia (3.2^0^/_0000_), Bulgaria (3.6^0^/_0000_), Romania (4.4^0^/_0000_), and Estonia (4.4^0^/_0000_) (Ferlay et al., 2018B). Still, there is an upward trend in incidence (T=+6.6%). A similar significant increase in incidence is observed in other developed countries where high public access to medical care is a major factor in the increase of detection. 

According to the studies aimed to assess the frequency of overdiagnosis, 228,000 TC cases (70-80%) registered in US women from 1998 to 2007 were asymptomatic and could remain unnoticed throughout life. Overdiagnosis could also be the case in the same period in 90% of female cases in South Korea, 70-80% of cases in Italy, France and Australia, 50% of cases in Japan, Scandinavian countries, England and Scotland, (Roman et al., 2017). In recent decades, the TC incidence of thyroid cancer was steadily and consistently growing in many developed countries. The most remarkable sevenfold growth was observed in South Korea – from 6.3 cases per 100,000 population in 1999 to 47.5 cases in 2009 (Park et al., 2016), and 60.70/0000 nowadays (Ferlay et al., 2018B). Some researchers attribute this fact to overdiagnosis, in particular, due to the extensive use of ultrasonography (Park et al., 2016). Previous studies in South Korea have shown a strong correlation between the TC incidence in 2009 and the screening coverage in 2008 and 2009 (Jung et al., 2012).

The component analysis has proven that the increase in the TC incidence in Kazakhstan was mainly caused by the increased disease risk (Δ_R_=+61.7%), while the share of various changes in the population structure was only 15.4%. Those phenomena could be associated with an increase in detection of new TC cases, including those without obvious clinical manifestations. In one study, a 97.1% increase in the estimated TC incidence was due to a more extensive detection of tumors at regional and localized stages characterized by 100% five-year relative survival rates (Park et al., 2016).

Thus, the TC incidence in Kazakhstan is repeating the global trend of the growth of indicators and changes in the contribution/evolution of risk factors affecting the development of this pathology, as evidenced by high growth rates in general and in different age groups.

Upward trends of TC incidence in Kazakhstan may be associated with growing diagnostic potential and increased availability of highly sensitive ultrasound methods. It sometimes leads to overdiagnosis and subsequent treatment (including surgery) of patients without any clinical manifestations. Overdiagnosis has adverse consequences both from the point of the patients and the socio-economic burden. Moreover, we should not exclude other possible reasons including risk factors for thyroid cancer such as benign thyroid gland diseases, family history, some intestine conditions (family adenomatous polyposis), acromegalia, benign mammary gland diseases, height and weight, exposure to radiation, reproductive history of women, and etc. The evolution of risk factors and the increase in the number of these causes are also the cause of the TC growth in Kazakhstan.

The component analysis can give answers to many questions, like the level of association of TC incidence with the population aging and the impact of new or intensified epidemiological factors on the disease risk. A possible impact of changes in the system of patients’ registration and in the quality of diagnostics shall be excluded. The current results can be the basis for further studies and are recommended for use for the planning of anticancer activities and the development of screening programs.
